# Traditional Chinese medicine in pancreatitis: mechanisms, integrative strategies, and clinical perspectives

**DOI:** 10.3389/fmed.2026.1796862

**Published:** 2026-04-30

**Authors:** Yinxiong He, Xinyue Sun, Kuanyu Wang

**Affiliations:** 1 Kunming Municipal Hospital of Traditional Chinese Medicine, Kunming, Yunnan, China; 2 Heilongjiang University of Chinese Medicine, Harbin, Heilongjiang, China; 3 First Affiliated Hospital, Heilongjiang University of Chinese Medicine, Harbin, Heilongjiang, China

**Keywords:** diagnosis, genetics, individualized treatment, pancreatitis, pathophysiology, TCM

## Abstract

Pancreatitis is a heterogeneous inflammatory disorder comprising both acute and chronic forms, with pathogenesis driven by interactions among genetic susceptibility, metabolic factors, inflammatory signaling, and disruption of intestinal barrier function. Despite advances in imaging and supportive care, its management remains largely non-specific, and effective targeted therapies are limited, particularly in severe disease and chronic progression. There is increasing evidence suggesting that traditional Chinese medicine (TCM) may provide complementary therapeutic value through multi-target regulation of key pathogenic processes, including inflammatory amplification, gut microbiota imbalance, microcirculatory disturbance, and fibrosis-related pathways. In this review, we synthesize recent advances in the epidemiology, molecular heterogeneity, diagnostic strategies, and pathophysiological mechanisms of pancreatitis, focusing on the mechanistic convergence between TCM interventions and modern biomedical pathways. In addition, we propose a phenotype-driven integrative framework which could be consider to identify patient subgroups likely to benefit from TCM-based therapies, including metabolically driven, microbiota-associated, and fibrosis-related phenotypes. Despite these, current evidence remains limited by methodological heterogeneity, small sample sizes, and lack of standardized outcome measures. Future research for improved alignment between TCM syndrome differentiation and objective biomarkers is needed, and a precision integrative approach may enhance therapeutic effectiveness and support more individualized management of pancreatitis.

## Introduction

Pancreatitis represents a heterogeneous inflammatory disorder encompassing acute pancreatitis (AP) and chronic pancreatitis (CP), both of which contribute substantially to global healthcare burden (1). Recent epidemiological analyses indicate that the incidence of AP remains high worldwide, with estimates ranging from 30 to 50 cases per 100,000 population annually, while CP continues to impose significant long-term morbidity due to irreversible structural damage and functional insufficiency of the pancreas (1, 2). Although advances in diagnostic imaging and supportive care have improved early recognition and short-term outcomes, pancreatitis remains associated with a significant rate of hospitalization, healthcare costs and disease recurrence, particularly in patients with metabolic risk factors such as obesity, hypertriglyceridemia and diabetes mellitus (2, 3). These underscore that pancreatitis is increasingly recognized not only as an acute inflammatory condition but also as a systemic and metabolically influenced disease entity.

Despite improvements in clinical management, several therapeutic challenges remain unresolved. For instance, severe AP (SAP) continues to carry a high risk of organ failure and mortality, driven by dysregulated systemic inflammation and multi-organ dysfunction, including acute lung injury and circulatory collapse (1, 4). In both AP and CP, disruption of the intestinal barrier and alterations in the gut microbiota have been implicated in disease progression, infectious complications, and systemic inflammatory amplification (5). Moreover, recurrent attacks, persistent abdominal pain, and progressive fibrosis in CP significantly impair quality of life and are inadequately addressed by current pharmacological and interventional strategies (2, 6). Notably, existing treatment paradigms remain largely supportive, with limited availability of targeted therapies capable of modulating key pathogenic pathways such as inflammation, microcirculatory disturbance, and immune dysregulation.

Increasing attention has been directed toward integrative therapeutic approaches, particularly those derived from traditional Chinese medicine (TCM), which are characterized by multi-target and system-level regulatory effects. Emerging evidence suggests that TCM interventions may exert beneficial effects in pancreatitis through modulation of inflammatory signaling pathways, restoration of intestinal barrier function, regulation of gut microbiota, and improvement of pancreatic microcirculation (7, 8). Furthermore, certain herbal formulations and acupuncture-based therapies have demonstrated potential in attenuating disease severity, reducing complications, and promoting functional recovery when used as adjuncts to conventional treatment (8, 9). However, the integration of TCM within modern pancreatitis management remains limited by fragmentation of mechanistic evidence, variability in clinical study quality, and insufficient alignment between traditional syndrome differentiation and contemporary biomedical frameworks.

Accordingly, this review aims to provide a focused synthesis of current evidence on pancreatitis from the perspective of TCM and integrative medicine, with particular emphasis on mechanistic convergence, clinical applicability, and future directions for precision-based therapeutic strategies.

## Molecular heterogeneity and integrative therapy

Pancreatitis exhibits substantial molecular heterogeneity arising from the interaction between genetic susceptibility and environmental exposures, which may influence both disease phenotype and therapeutic responsiveness. Among the most consistently implicated pathways is dysregulated intrapancreatic trypsin activation, driven by variants in genes such as PRSS1 and SPINK1 (10, 11). Gain-of-function mutations in PRSS1 promote premature trypsinogen activation, whereas loss-of-function variants in SPINK1, a key trypsin inhibitor, reduce protective capacity against protease-mediated injury, thereby predisposing to recurrent and chronic pancreatic inflammation (10, 11). Clinically, these variants have been associated with earlier disease onset, increased recurrence, and a higher risk of progression to CP, suggesting a subgroup characterized by sustained enzymatic injury and inflammatory amplification (11). From an integrative perspective, such patients may represent a phenotype in which interventions targeting inflammatory cascades, oxidative stress, and cellular injury, which are mechanisms frequently implicated in TCM pharmacology, are particularly relevant.

In parallel, abnormalities in pancreatic ductal secretion and fluid homeostasis, primarily mediated by CFTR variants, contribute to impaired bicarbonate secretion, increased ductal viscosity and subsequent ductal obstruction (12), and are relevant in idiopathic and recurrent pancreatitis, where altered ductal microenvironment promotes both inflammation and fibrosis. Notably, emerging evidence suggests that pancreatic ductal dysfunction is closely linked to gut-pancreas axis disturbances, including microbiota alterations and intestinal barrier impairment (13). Given that several TCM formulations have been shown to modulate gut microbiota composition and enhance intestinal barrier integrity, this subgroup may exhibit differential responsiveness to microbiota-targeted integrative therapies.

Variants in CTRC, which regulates trypsin degradation, further contribute to disease susceptibility by impairing protective proteolytic pathways, thereby amplifying intrapancreatic protease activity (11). Beyond protease-related mechanisms, recent studies have emphasized the role of inflammatory and immune regulatory pathways, including NF-κB and inflammasome activation, in mediating disease severity and systemic complications (14). These pathways not only represent central drivers of pancreatic injury but also constitute key targets of several TCM-derived compounds and formulations, which have demonstrated inhibitory effects on pro-inflammatory cytokine release and immune dysregulation in experimental and clinical settings (7).

Taken together, these molecular pathways highlight that pancreatitis is not a uniform disease entity but rather comprises biologically distinct subgroups defined by protease dysregulation, ductal dysfunction, and immune activation. Although current clinical practice does not routinely stratify patients based on genetic background, such molecular heterogeneity provides a conceptual basis for precision integrative approaches. Future studies integrating genetic profiling with clinical phenotypes and treatment response data may help identify patient subsets that derive greater benefit from TCM-based adjunctive therapies, particularly those targeting inflammation, microcirculation, and gut barrier function.

## Diagnosis

### Imaging and clinical stratification

The diagnostic evaluation of pancreatitis is based on the integration of clinical manifestations, laboratory findings, and imaging modalities; however, for integrative medicine, the main objective of diagnosis extends beyond confirmation of disease toward risk stratification and identification of therapeutic targets relevant to adjunctive interventions. Contrast-enhanced computed tomography (CECT) remains the standard imaging modality for assessing pancreatic necrosis and local complications, while its severity grading correlates with clinical outcomes, including organ failure and mortality (15). In parallel, imaging findings such as pancreatic edema, peripancreatic fluid collection and necrosis distribution provide indirect indicators of inflammatory burden and microcirculatory impairment, which are key therapeutic targets in TCM.

From a clinical perspective, early severity assessment using scoring systems such as the bedside index for severity in acute pancreatitis (BISAP) and Acute Physiology and Chronic Health Evaluation II (APACHE II) is essential for identifying high-risk patients, with elevated scores being associated with increased incidence of systemic inflammatory response syndrome and multi-organ dysfunction (16). These indices are not only prognostic tools but are also frequently used as objective outcome measures in integrative clinical studies, particularly in evaluating interventions aimed at reducing systemic inflammation and improving organ function.

### Biomarkers and therapeutic monitoring

Recent advances in biomarker research have enhanced the capacity for early risk prediction and dynamic monitoring of pancreatitis. Inflammatory markers, including C-reactive protein (CRP), interleukin-6 (IL-6), and procalcitonin, demonstrate strong associations with disease severity and progression (17). Additionally, the neutrophil-to-lymphocyte ratio (NLR) has emerged as a simple yet effective indicator of systemic inflammation, with elevated levels correlating with severe disease and prolonged hospitalization (18). They have become relevant in TCM, as many therapeutic formulations are proposed to exert anti-inflammatory and immunomodulatory effects, and their clinical efficacy is often reflected by reductions in these indices.

Furthermore, increasing attention has been directed toward biomarkers associated with intestinal barrier dysfunction and microbiota imbalance. Disruption of the gut barrier facilitates bacterial translocation and amplifies systemic inflammation, thereby contributing to disease severity (19), and this mechanism aligns closely with TCM concepts such as damp-heat accumulation (a pathological state characterized by inflammatory and metabolic disturbance) and spleen deficiency (functional impairment of digestion and barrier regulation), providing a potential bridge between traditional syndrome differentiation and measurable biomedical parameters.

### Integration with TCM syndrome differentiation

In TCM clinical practice, diagnosis is guided by syndrome differentiation (pattern identification based on symptom clusters and functional imbalance), which directs individualized treatment strategies. AP is commonly associated with patterns such as liver-qi stagnation (functional disturbance of energy flow leading to pain and stress-related exacerbation) and damp-heat accumulation (inflammatory and metabolic overload), whereas CP is more frequently characterized by spleen deficiency and blood stasis (chronic circulatory impairment and fibrosis tendency) (20). Emerging studies suggest that these patterns may correspond to distinct biological profiles, including differences in inflammatory markers, metabolic parameters, and gastrointestinal function (7).

The integration of conventional diagnostic frameworks with syndrome differentiation offers a potential pathway toward standardized and reproducible integrative diagnosis (21). By correlating objective indicators such as inflammatory burden, microcirculatory status, and intestinal function with TCM patterns, clinicians may better identify patient subgroups that are more likely to benefit from specific adjunctive therapies. This approach also facilitates the incorporation of measurable endpoints into clinical studies, thereby improving the interpretability and comparability of TCM-based interventions.

## Pathophysiological mechanism

### Inflammatory amplification and immune dysregulation

Inflammatory amplification represents the central driver of disease progression in pancreatitis and a primary therapeutic target of TCM. The early activation of pattern recognition receptors can trigger downstream signaling cascades, particularly NF-κB and NLRP3 inflammasome pathways, resulting in excessive production of proinflammatory cytokines, including IL-1β, IL-6, and TNF-α, which further propagate systemic inflammatory response and multi-organ dysfunction (22–24). Recent studies have emphasized the role of innate immune reprograming, including neutrophil extracellular trap (NET) formation and macrophage polarization, in sustaining inflammatory amplification and tissue injury (23, 25).

Based on such a framework, TCM interventions characterized by heat-clearing and detoxifying effects (therapeutic approach aimed at suppressing excessive inflammatory and metabolic activity) have shown potential to attenuate inflammatory cascades through multi-target modulation. For example, emodin (a bioactive anthraquinone compound derived from medicinal herbs) has been shown to inhibit NLRP3 inflammasome activation and reduce IL-1β maturation, thereby limiting systemic inflammatory amplification (26). Similarly, Qingyi Decoction (a herbal formula used to clear heat and resolve toxicity) has been shown to suppress NF-κB signaling and reduce circulating inflammatory mediators, aligning mechanistically with anti-inflammatory strategies (27).

Importantly, emerging evidence suggests that TCM may also modulate immune cell phenotypes, shifting macrophage polarization from a proinflammatory M1 state toward an anti-inflammatory M2 phenotype (immune-regulatory state promoting tissue repair), thereby restoring immune homeostasis rather than inducing broad immunosuppression (25, 28). This bidirectional regulation distinguishes TCM-based approaches from single-target pharmacologic interventions.

### Intestinal barrier dysfunction and microbiota imbalance

Disruption of the intestinal barrier is increasingly recognized as a pivotal contributor to disease severity in pancreatitis. Increased intestinal permeability facilitates bacterial translocation and endotoxin release, which further amplify systemic inflammation via toll-like receptor signaling and inflammasome activation (29, 30). Concurrently, dysbiosis characterized by reduced beneficial commensals and overgrowth of pathogenic bacteria contributes to metabolic endotoxemia and immune dysregulation (9, 10). From a TCM perspective, this pathological process corresponds to dysfunction of digestive and barrier regulation (conceptually described as impaired transformation and transport function), providing a rationale for interventions targeting the gut-pancreas axis. It has been demonstrated that several TCM formulations can restore intestinal barrier integrity by upregulating tight junction proteins, including occludin and zonula occludens-1, while simultaneously modulating microbiota composition (31, 32).

The Qingyi Decoction has been shown to increase short-chain fatty acid-producing bacteria (microbial metabolites with anti-inflammatory and barrier-protective effects), thereby activating AMPK-dependent pathways and suppressing inflammatory signaling (32). Dachengqi Decoction (a purgative formula used to eliminate internal accumulation) improves intestinal motility and reduces endotoxin translocation, indirectly attenuating systemic inflammation (33). These findings suggest that TCM interventions may exert therapeutic effects through coordinated regulation of microbiota composition, barrier integrity, and immune signaling, rather than acting solely at the pancreatic level.

### Microcirculatory disturbance and tissue hypoxia

Microcirculatory impairment is a hallmark of severe pancreatitis and contributes to pancreatic necrosis and organ dysfunction. Endothelial injury, increased vascular permeability, leukocyte adhesion, and microthrombus formation collectively result in reduced tissue perfusion and hypoxia, thereby exacerbating cellular injury (34, 35). Recent imaging and experimental studies have demonstrated that microvascular dysfunction occurs early in disease progression and correlates with necrotic transformation and clinical severity (35).

TCM interventions targeting blood stasis (pathological state characterized by impaired circulation and microvascular obstruction) have shown potential in improving microcirculatory dynamics. Xuefu Zhuyu Decoction (a classical formula used to promote blood circulation and remove stasis) has been reported to enhance pancreatic perfusion, reduce vascular resistance, and improve microvascular blood flow parameters in both experimental and clinical settings (36). Mechanistically, these effects are associated with inhibition of endothelial adhesion molecules and reduction of oxidative stress-mediated vascular injury (37).

In addition, bioactive compounds such as salvianolic acid (a phenolic compound derived from medicinal herbs) have been shown to protect endothelial function by modulating nitric oxide bioavailability and reducing oxidative stress, thereby restoring microvascular integrity (37). These findings indicate that TCM-based therapies may directly target the microcirculatory component of pancreatitis, which is often insufficiently addressed by conventional supportive treatments.

### Acinar cell death, autophagy dysfunction, and mitochondrial stress

Acinar cell injury represents the initiating event in pancreatitis, characterized by intracellular calcium overload, premature trypsinogen activation, and mitochondrial dysfunction. Recent studies have highlighted the important role of impaired autophagy and defective mitophagy in amplifying cellular injury, leading to accumulation of damaged organelles and activation of inflammatory signaling pathways (38, 39). Mitochondrial permeability transition and ATP depletion further exacerbate necrotic cell death and inflammatory amplification (39). TCM interventions have demonstrated regulatory effects on these intracellular processes. For instance, emodin has been shown to restore autophagic flux (cellular degradation pathway responsible for removing damaged organelles) and improve mitochondrial function by reducing oxidative stress and stabilizing mitochondrial membrane potential (40). Similarly, baicalein (a flavonoid compound derived from medicinal herbs) has been reported to attenuate calcium overload and inhibit mitochondrial-mediated apoptosis pathways (41).

Dachaihu Decoction (a formula used to harmonize internal organ function and reduce inflammation) has been shown to modulate MAPK signaling and reduce acinar cell apoptosis, thereby limiting pancreatic injury progression (20). These findings suggest that TCM therapies may act at the level of intracellular stress responses, targeting key processes such as autophagy, mitochondrial integrity, and programed cell death.

### Fibrosis progression and pain sensitization

CP is characterized by progressive fibrosis and persistent abdominal pain, which are driven by activation of pancreatic stellate cells (PSCs) and neuroimmune interactions (42). Transforming growth factor-β (TGF-β) signaling plays a central role in PSC activation and extracellular matrix deposition, leading to irreversible structural remodeling. In parallel, chronic inflammation induces peripheral and central sensitization, contributing to persistent pain even after resolution of acute inflammation (43).

TCM interventions targeting blood stasis (circulatory impairment and fibrotic tendency) and qi stagnation (functional dysregulation associated with pain and impaired flow) have demonstrated antifibrotic and analgesic effects. Experimental studies have shown that herbal formulations can inhibit TGF-β/Smad signaling and reduce collagen deposition, thereby attenuating fibrotic progression (44, 45). Additionally, acupuncture (a neuromodulatory intervention involving stimulation of specific points) has been reported to modulate central pain pathways and reduce pain perception through activation of endogenous opioid and vagal anti-inflammatory pathways (46).

Recent evidence also suggests that TCM may influence neuroimmune interactions by regulating cytokine-mediated neuronal sensitization, thereby providing a mechanistic basis for its role in chronic pain management (47). This integrative effect on both structural remodeling and pain signaling pathways highlights the potential of TCM in addressing long-term disease burden in CP.

## Mechanistic convergence between TCM and modern pancreatitis biology

### TCM regulation of the gut-pancreas axis

The gut-pancreas axis has emerged as a central component in pancreatitis progression, linking intestinal dysbiosis, barrier dysfunction, and systemic inflammation. Experimental and clinical studies have shown that disruption of gut microbiota contributes to endotoxemia and inflammatory amplification in AP (48, 49). Recent evidence indicates that TCM formulations such as Qingyi Decoction (a heat-clearing and detoxifying formula) modulate gut microbial composition and restore metabolic homeostasis, including short-chain fatty acid production, which may attenuate systemic inflammatory responses in severe AP (7). In addition, microbiota-targeted effects of TCM have been associated with reduced intestinal permeability and decreased bacterial translocation, suggesting that TCM interventions may act upstream of pancreatic inflammation by stabilizing host-microbiota interactions (7).

### TCM effects on microcirculation

Microcirculatory disturbance is an important determinant of pancreatic necrosis and organ failure, characterized by endothelial dysfunction, leukocyte adhesion, and microvascular thrombosis (15). TCM approaches targeting blood stasis (a pathological state characterized by impaired circulation and microvascular obstruction) have shown measurable effects on pancreatic perfusion and vascular dynamics. Systematic analyses indicate that TCM formulations can improve microcirculatory parameters and reduce vascular resistance in AP (34). Mechanistically, these effects are linked to modulation of endothelial function and attenuation of oxidative stress, which are key drivers of microvascular injury in pancreatitis (15, 34).

### TCM modulation of NLRP3, NF-κB, MAPK, and JAK/STAT signaling

Inflammatory signaling pathways, particularly NLRP3 inflammasome activation and NF-κB signaling, play central roles in cytokine release and disease severity in pancreatitis (50). Recent studies have shown that Qingyi Decoction (a detoxifying anti-inflammatory formula) suppresses NLRP3 inflammasome activation and downstream IL-1β production, thereby reducing pancreatic and systemic inflammation (51). Natural compounds derived from TCM, including polyphenols and flavonoids (bioactive plant-derived molecules), have also been reported to inhibit NF-κB and MAPK signaling pathways, leading to reduced inflammatory cell infiltration and tissue injury (52). Furthermore, honokiol (a bioactive lignan compound) has been shown to attenuate intestinal and systemic inflammation by inhibiting the JAK/STAT signaling pathway, highlighting the ability of TCM-derived compounds to regulate multiple inflammatory axes simultaneously (53).

### TCM and intestinal barrier protection

Intestinal barrier dysfunction is a key contributor to disease progression in severe AP, facilitating bacterial translocation and systemic inflammatory response syndrome (49). Da-Cheng-Qi Decoction (a formula used to restore intestinal motility and eliminate accumulation) has been shown to preserve intestinal barrier integrity and reduce HMGB1-mediated inflammatory signaling, thereby limiting progression to organ failure (54). Similarly, experimental studies demonstrate that TCM interventions enhance tight junction protein expression and reduce epithelial permeability, supporting their role in maintaining mucosal barrier function during pancreatitis (53, 54). These findings indicate that TCM therapies may act at the level of intestinal barrier preservation, which is a critical but often under-targeted component of pancreatitis management. Taken together, these findings demonstrate that TCM interventions may be used to converge with modern pancreatitis biology at several key mechanistic nodes, including gut microbiota regulation, microcirculatory improvement, inflammatory signaling suppression, intestinal barrier preservation, and organ cross-talk modulation. Rather than acting as isolated symptomatic therapies, TCM approaches appear to exert multi-target effects across interconnected biological systems, aligning with current concepts of pancreatitis as a systemic and network-based disease (48, 50). This convergence provides a rationale for integrating TCM into mechanistically informed therapeutic strategies, particularly in severe disease where conventional supportive care does not directly address these underlying pathways.

### Precision integrative medicine: who may benefit most from TCM?

AP is increasingly recognized as a biologically heterogeneous condition with distinct etiological and mechanistic subtypes, which provides a rationale for phenotype-driven integrative treatment strategies (15, 55). Patients with hypertriglyceridemic AP represent a metabolically driven subgroup characterized by lipotoxicity, oxidative stress, and amplified inflammatory responses, in which multi-target interventions may be particularly relevant. Experimental evidence indicates that bioactive compounds such as quercetin (a plant-derived flavonoid with anti-inflammatory properties) can attenuate lipid-induced pancreatic injury and reduce cytokine release in this context (56). In biliary AP, where transient obstruction and bile reflux trigger localized pancreatic inflammation, supportive strategies that promote gastrointestinal motility and reduce inflammatory mediator accumulation may complement standard management, although robust clinical evidence for integrative approaches remains limited (15). In severe AP complicated by acute lung injury, systemic inflammatory amplification and gut-lung axis interactions are central, and recent studies demonstrate that Qingyi Decoction (a formula targeting intestinal and inflammatory regulation) can modulate microbiota-derived metabolites and suppress AMPK/NF-κB/NLRP3 signaling, thereby attenuating both pancreatic and pulmonary injury (7).

Emerging data support the existence of mechanistic phenotypes that may guide integrative interventions. Patients with gut dysbiosis-dominant AP exhibit altered microbial composition and impaired intestinal barrier function, which contribute to endotoxemia and systemic inflammation; TCM interventions have been shown to restore microbial balance and enhance short-chain fatty acid-mediated signaling pathways in experimental and translational settings (7, 57, 58). Individuals with an inflammatory-high phenotype, characterized by excessive activation of pathways such as NLRP3 inflammasome and NF-κB signaling, may benefit from multi-target anti-inflammatory modulation, as highlighted in recent mechanistic studies of TCM compounds (26). In recurrence-prone AP, persistent metabolic or microbiota-related dysregulation may underlie repeated disease episodes, suggesting a potential role for interventions that restore systemic homeostasis, although longitudinal evidence remains limited (55). Finally, in CP, progressive fibrosis and persistent pain are driven by pancreatic stellate cell activation and neuroimmune interactions, and TCM approaches targeting blood stasis (microcirculatory impairment and fibrotic tendency) and qi stagnation (functional dysregulation associated with pain) may exert antifibrotic and analgesic effects through modulation of fibrogenic signaling and neuronal sensitization pathways (42).

A structured overview of these phenotype-mechanism-intervention relationships is provided in Table 1, which summarizes the key clinical subgroups, dominant biological features, and corresponding TCM intervention targets. Importantly, this phenotype-oriented framework shifts the role of TCM from a generalized adjunctive therapy toward a stratified, mechanism-informed approach. Rather than applying uniform interventions across all patients, this model supports selective integration of TCM based on dominant pathophysiological drivers, which is more consistent with current precision medicine principles. However, this approach remains largely hypothesis-driven, and its clinical applicability will depend on future studies that prospectively validate phenotype-based treatment selection and establish standardized criteria linking TCM pattern differentiation (individualized functional classification system) with reproducible biomedical markers.

**TABLE 1:** Phenotype-driven integrative application of TCM in pancreatitis.

Phenotype/ subgroup	Key mechanistic features	Potential TCM targets (with explanation)	Representative mechanistic effects	Evidence level	Key references
Hypertriglyceridemic acute pancreatitis	Lipotoxicity, oxidative stress, cytokine amplification	Heat-toxin accumulation (inflammatory excess); phlegm-dampness (lipid metabolic dysregulation)	Reduction of inflammatory cytokines; modulation of lipid-induced injury	Preclinical	(56)
Biliary AP	Obstruction-induced inflammation, bile reflux	Stagnation resolution (functional obstruction and impaired flow)	Promotion of gastrointestinal motility; reduction of inflammatory mediators	Limited clinical	(15)
Severe acute pancreatitis with ALI	Systemic inflammation, gut–lung axis activation	Detoxification (systemic inflammatory clearance); gut regulation (microbiota stabilization)	Modulation of AMPK/NF-κB/NLRP3 pathway; microbiota-derived metabolite regulation	Preclinical + emerging clinical	(7)
Gut dysbiosis–dominant phenotype	Microbiota imbalance, barrier dysfunction	Intestinal regulation (restoration of microbiota and barrier integrity)	Increased short-chain fatty acid signaling; reduced endotoxemia	Preclinical + translational	(7, 57, 58)
Inflammatory-high phenotype	NLRP3, NF-κB pathway activation	Heat-clearing (anti-inflammatory regulation)	Suppression of inflammasome activation and cytokine release	Preclinical	(26)
Recurrence-prone phenotype	Persistent metabolic/microbiota dysregulation	Systemic regulation (restoration of immune–metabolic balance)	Modulation of immune homeostasis; reduction of relapse drivers	Limited clinical	(55)
Chronic pain/fibrosis phenotype	PSC activation, fibrosis, neuroimmune sensitization	Blood stasis (microcirculatory impairment/fibrosis); qi stagnation (pain-related dysfunction)	Inhibition of fibrogenic signaling; modulation of neuroimmune pathways	Preclinical + limited clinical	(42)

### Evidence and methodological limitations of TCM studies in pancreatitis

Current evidence on TCM in pancreatitis most consists of preclinical studies and small-scale clinical investigations, with relatively few high-quality randomized controlled trials (RCTs). Experimental studies have shown that TCM interventions can regulate inflammatory signaling, intestinal barrier function, and microcirculation; however, these findings are largely derived from animal models and cannot directly establish clinical efficacy (15, 59). Clinical evidence has increased in recent years, including randomized studies of specific formulas such as Chaiqinchengqi Decoction (a compound formula used to regulate intestinal function and reduce inflammation) and trials of electroacupuncture (a neuromodulatory intervention using electrical stimulation through acupuncture needles), but most studies remain single-center with limited sample sizes and short follow-up durations, restricting generalizability and long-term interpretation (7, 56). In addition, real-world studies suggest potential benefits of integrative TCM and Western treatment in reducing inflammation and hospital stay, but these observational designs are inherently subject to confounding and cannot replace rigorously controlled trials (5).

Several methodological limitations further affect the reliability and comparability of existing studies. First, syndrome differentiation (pattern-based classification used to guide individualized TCM treatment) is inconsistently defined across studies, making it difficult to standardize patient selection or link TCM patterns with biomedical phenotypes (26). Second, substantial heterogeneity exists in treatment protocols, including differences in herbal composition, dosage, treatment duration, and frequent use of co-interventions such as enemas, external therapies, or concurrent pharmacological treatment, which complicates attribution of therapeutic effects (42). Third, placebo control and blinding are often absent due to the sensory characteristics of herbal formulas and procedural interventions, increasing the risk of bias in subjective outcomes such as pain and symptom relief (7). Fourth, outcome measures are not standardized, with studies variably reporting biochemical indices, symptom scores, or composite “effective rates,” limiting cross-study comparison and meta-analysis (5). Finally, multicenter validation remains scarce despite increasing publication volume, and most findings have not been reproduced across diverse populations or clinical settings (26). Together, these limitations indicate that while the current evidence base is mechanistically informative and clinically suggestive, it remains methodologically heterogeneous and requires more rigorously designed multicenter trials with standardized diagnostic criteria and outcome measures.

## Future perspectives

Future research in pancreatitis could move beyond descriptive and single-mechanism studies toward integrated, clinically oriented investigation. An important priority is the establishment of standardized frameworks linking TCM syndrome differentiation (pattern-based classification guiding individualized treatment) with measurable biomedical parameters, including inflammatory markers, microbiota profiles, and imaging-based severity indices. Such integrations would allow a more consistent patient stratification and improve reproducibility across studies. In parallel, the development of multicenter randomized controlled trials with clearly defined inclusion criteria, standardized treatment protocols, and unified outcome measures is essential to strengthen the clinical evidence base and reduce current heterogeneity.

Since pancreatitis comprises distinct subgroups defined by metabolic disturbance, inflammatory burden, microbiota imbalance and fibrosis progression, future studies prospectively evaluating whether specific TCM interventions demonstrate differential efficacy across these phenotypes, rather than applying uniform treatment approaches is needed. In this context, incorporation of multi-omics techniques, including genomics, metabolomics, and microbiome analysis, may help identify biomarkers associated with treatment response and facilitate more targeted therapeutic strategies. Additionally, further investigation into the gut-pancreas axis, microcirculatory regulation and immune modulation may clarify how TCM interventions exert system-level effects in severe disease.

From a translational perspective, more efforts are needed for improving the standardization and quality control of TCM formulations. Variability in herbal composition, preparation methods, and dosing remains a major barrier to reproducibility and wider clinical adoption. The development of well-characterized formulations, alongside pharmacokinetic and pharmacodynamic studies, will be necessary to support regulatory acceptance and integration into evidence-based practice. Lastly, closer collaboration between clinicians, pharmacologists, and basic scientists is required to design studies that simultaneously address mechanism, efficacy, and safety, thereby bridging the gap between experimental findings and real-world clinical application.

## Conclusion

Pancreatitis is a complex and heterogeneous disease in which inflammatory amplification, intestinal barrier dysfunction, microcirculatory disturbance, and progressive fibrosis interact to determine clinical outcomes. Despite advances in supportive care, current treatment strategies remain largely non-specific and do not directly target many of these key pathogenic processes. Within this context, TCM offers a complementary approach characterized by multi-target regulation of interconnected biological pathways, including inflammatory signaling, gut microbiota, and microvascular function.

The evidence summarized in this review indicates that TCM interventions may provide clinically relevant benefits in selected patient populations, particularly when aligned with dominant pathophysiological features. However, the current evidence base remains limited by methodological heterogeneity, small sample sizes, and insufficient standardization, which restricts definitive conclusions regarding efficacy. The integration of TCM into modern pancreatitis management therefore requires a transition from empirically driven application toward mechanism-based and phenotype-oriented strategies.

In conclusion, the future role of TCM in pancreatitis lies not in its use as a generalized adjunct, but in its incorporation into a precision integrative framework guided by disease heterogeneity and biological targets. Achieving this will depend on rigorous clinical validation, improved methodological consistency, and closer alignment between traditional diagnostic concepts and contemporary biomedical markers. Such an approach has the potential to enhance therapeutic effectiveness and contribute to more individualized and comprehensive management of pancreatitis.
